# Rifaximin Is Effective for the Treatment of *Clostridium difficile*—Associated Diarrhea: Results of an Open-Label Pilot Study

**DOI:** 10.1155/2011/106978

**Published:** 2011-11-09

**Authors:** David T. Rubin, Sunana Sohi, Matthew Glathar, Tojo Thomas, Nicole Yadron, Bonnie L. Surma

**Affiliations:** ^1^University of Chicago Medical Center, Chicago, IL 60637, USA; ^2^Section of Gastroenterology, Hepatology and Nutrition, University of Chicago Medical Center, Chicago, IL 60637, USA

## Abstract

*Objectives*. This open-label trial assessed the efficacy and safety of rifaximin as first-line therapy in hospitalized patients with *Clostridium difficile*-associated diarrhea (CDAD). *Methods*. We enrolled thirteen patients who had a confirmed diagnosis of CDAD characterized by ≥3 unformed stools/day and positive *C. difficile* toxin assay. Those patients received rifaximin 400 mg three times daily for 10 days. Resolution of symptoms, repeat assay 10 days after treatment, and followup for recurrence were assessed. *Results*. Eight patients completed the study, and all reported symptom resolution during treatment. Mean time to last unformed stool was 132 h ± 42.5 h. Seven patients had no relapse by week 2 and in longer followup (median 162 days). One patient had recurrent CDAD during a repeat hospitalization. *Conclusions*. Rifaximin was effective and safe as first-line treatment for CDAD and did not result in recurrence in most patients.

## 1. Introduction

*Clostridium difficile *infection is one of the most common causes of nosocomial diarrhea and may be associated with substantial morbidity and mortality [1–3]. The prevalence and severity of *C. difficile*-associated diarrhea (CDAD) have been increasing since the mid-1990s [4, 5]. These epidemiologic changes may be related to the emergence of virulent, epidemic strains of *C. difficile*, particularly strains with binary toxin genes characterized as BI/NAP1, that have been implicated in multiple CDAD outbreaks in North America and Europe [3, 6, 7]. These previously uncommon *C. difficile *strains produce substantially higher levels of toxins A and B than other hospital-acquired *C. difficile *strains [7], are highly resistant to fluoroquinolone antibiotics [3, 6, 8], and may be associated with increased disease severity and mortality [3, 9]. 

Metronidazole and vancomycin are most commonly administered for the treatment of CDAD and represent the current standard of care [5, 10]. Metronidazole is generally considered first-line therapy [10, 11]. However, in addition to having the potential for adverse effects (e.g., nausea, vomiting, metallic taste), metronidazole is almost completely absorbed in the intestine and is present in low and variable levels in feces, suggesting that it may have limited direct action in the colon [10–12]. Because vancomycin is more expensive than metronidazole and may promote the growth of vancomycin-resistant enterococci, it is usually reserved for the treatment of severe CDAD or use in certain patient populations, such as pregnant women and those who are unresponsive to or unable to tolerate oral metronidazole [1, 11]. Furthermore, CDAD symptoms are unresponsive to metronidazole or vancomycin in as many as 25% of treated patients [13, 14], and up to 29% of patients experience symptom recurrence after initial successful treatment with either of these agents [14, 15]. Recurrence normally occurs within 2 weeks after completion of treatment. These data, along with reports of increasing recurrence rates after treatment with metronidazole or vancomycin in the presence of decreasing bacterial susceptibility to these antibiotics [16, 17], have prompted investigation of new therapies for CDAD.

Rifaximin is a rifamycin derivative characterized by a broad antimicrobial spectrum; it has activity against most Gram-negative and Gram-positive bacteria, as well as anaerobes and aerobes [18, 19]. When administered orally, rifaximin is virtually nonabsorbed (<0.4%) and exerts activity almost entirely within the intestinal lumen [20]. In vitro studies have shown rifaximin to have high levels of activity against multiple *C. difficile *isolates, including recent epidemic strains, with a 90% minimum inhibitory concentration of 0.015 *μ*g/mL and relatively low incidence of spontaneous resistance to *C. difficile* [21, 22]. Rifaximin has been extensively studied in numerous countries for the treatment of various conditions, including acute bacterial diarrhea, intestinal bacterial overgrowth, and hepatic encephalopathy, and is licensed in the United States for the treatment of travelers' diarrhea caused by noninvasive strains of *Escherichia coli *[19, 23]. 

Several small clinical studies with ≤8 patients [24, 25] and single-patient case reports [26, 27] have provided encouraging results using rifaximin for treating recurrent or refractory CDAD and preventing recurrence after successful treatment with vancomycin. However, data regarding the potential efficacy of rifaximin as initial therapy for CDAD are limited. A randomized study with 20 patients who received rifaximin 600 mg/d or vancomycin 1 g/d for 10 days demonstrated rifaximin to be as effective as vancomycin for resolving diarrhea [28]. An industry-sponsored larger prospective trial of rifaximin for CDAD (NCT 00269399) was discontinued due to difficulties with enrollment. This prospective, open-label study evaluated the efficacy and safety of rifaximin as a first-line treatment for CDAD in hospitalized patients in a university hospital setting.

## 2. Methods

This open-label pilot study was conducted at a single academic medical center of 400 beds and approximately 5–12 inpatient *C. difficile* tests ordered per day (source, personal communications: University of Chicago Infection Control Office and Microbiology Laboratory). Consecutive hospitalized patients >18 years of age with *C. difficile *infection were identified by referral from attending physicians or the hospital microbiology laboratory between January 2006 and October 2006 (a predefined study recruitment duration). Eligible patients had acute diarrhea (defined as ≥3 unformed stools during a 24-hour period) and a stool enzyme immunoassay (EIA) result positive for *C. difficile *toxin A or B. Patients were excluded if they had inflammatory bowel disease or microscopic colitis; symptoms suggesting moderate or severe dehydration (e.g., orthostasis); symptoms associated with fulminant colitis or toxic megacolon, including colon distention >10 cm on radiograph, peritoneal signs of rebound, or abdominal guarding due to colitis; received metronidazole or oral vancomycin within 2 days before recruitment or received >2 doses of an antidiarrheal agent within 8 hours before recruitment. Patients with feeding tubes and female patients with no menses for greater than 14 days prior to enrollment were also excluded. All patients provided written informed consent before receiving study treatment. The study protocol and informed consent form were approved by the University of Chicago Institutional Review Board. 

Patients who met inclusion criteria received oral rifaximin (Xifaxan; Salix Pharmaceuticals, Inc, Morrisville, NC) 400 mg three times daily for 10 days. Patients were instructed to complete daily entries on a stool diary card to document the frequency and consistency of bowel movements during the treatment period. Stools were characterized as formed if they retained their shape or unformed if they took the shape of their container or could be poured. Patients recorded in a stool diary the date, time, and stool consistency after each bowel movement. During the treatment period, patients were visited daily while in the hospital or telephoned at home after discharge to ensure protocol compliance and monitor for adverse events. Stool samples for repeat EIA analysis were collected 10 days after rifaximin treatment. Patients were contacted via telephone 2 weeks after completion of rifaximin therapy and up to 180 days later per protocol to assess short- and long-term symptom relapse, defined as ≥3 unformed stools per day. 

The primary endpoint was time from ingestion of the first dose of rifaximin to symptom resolution. Secondary endpoints included time to last unformed stool (TLUS), number of treatment failures, eradication of toxin in the stool as determined by negative stool EIA result immediately after rifaximin treatment, relapse rates, and adverse events.

## 3. Results

Thirteen consecutive hospitalized patients with CDAD who met eligibility criteria were enrolled and began treatment with rifaximin per protocol. Five of these 13 patients did not complete the treatment course and were excluded from analysis: 1 patient developed hypercapnic respiratory failure unrelated to the study treatment, 1 patient voluntarily withdrew after transfer to an extended care facility, and 3 patients violated protocol. Of the protocol violations, two did not take rifaximin as instructed and 1 subject was lost to followup despite numerous attempts to contact her. None were due to side effects during the time on observed therapy. 

Eight patients (mean age, 55 y; range, 39–76 y) completed the protocol and received rifaximin 1200 mg/d for 10 days (Table 1). Of these 8 patients, 7 had no prior history of CDAD. The patient with previous history of CDAD had a history of endometrial cancer and was receiving chemotherapy. She had three recurrences of CDAD before hospitalization and was symptomatic upon admission; prior treatments included metronidazole and vancomycin.

All of the 8 patients who completed rifaximin treatment achieved symptom resolution, with an overall mean TLUS of 151 hours (range, 84–282 h; Table 2). Of these 8 patients, 7 (88%) had symptom resolution during the prespecified 10-day treatment period (primary endpoint). The mean TLUS of these 7 patients was 132 h (range, 84–192 h). One patient with endometrial cancer completed 10 days of treatment but achieved symptom resolution at day 12 of followup. 

 Rifaximin was well tolerated, with no drug-related adverse events reported during the rifaximin treatment period. 

Stool samples for followup analysis were available for 7 patients; 5 of these 7 patients had stool EIA results negative for *C. difficile* toxin 10 days after initiating rifaximin treatment. One of the remaining patients who had positive stool toxin results on day 10 had achieved symptom resolution 4 days after beginning rifaximin treatment and remained symptom-free through the end of treatment; further treatment was not administered, since symptom resolution was achieved. The other patient was the woman with endometrial cancer. In subsequent followup, and while receiving chemotherapy, she was symptomatic and remained positive for EIA for *C. difficile*. At this point, oral vancomycin therapy was provided for 14 days. In the 49-day followup for this patient, she had become asymptomatic. 

Of the 7 patients who achieved symptom resolution within 10 days of rifaximin therapy, none had symptom recurrence within 2 weeks after treatment, including the patient who was asymptomatic despite positive stool toxin results at the end of rifaximin treatment. The median long-term followup available for the 7 patients who responded to rifaximin treatment was 160 days (range, 36–261 d). During this period, 1 of these 7 patients experienced CDAD recurrence. This patient developed symptoms at followup day 50, most likely as a complication of a prolonged hospitalization for end-stage heart failure; CDAD was confirmed by stool EIA on day 52 and was treated with metronidazole.

## 4. Discussion

In this current prospective, open-label study, rifaximin 1200 mg/d for 10 days demonstrated a favorable safety profile and was an effective initial therapy for CDAD in hospitalized patients. All of the 8 patients included had symptom resolution during the course of rifaximin treatment; 6 patients had eradication of *C. difficile *stool toxins. In all patients, no drug-related adverse events were reported. The rifaximin success rate in this study (86%) was similar to rates reported in studies of vancomycin and metronidazole [11, 13, 16, 29]. 

The findings presented in this study, including the observation that 1 patient achieved symptom resolution despite persistence of stool *C. difficile *toxin, are corroborated by a 1990 randomized, open-label study in which rifaximin 600 mg/d administered for 10 days resolved diarrheal symptoms associated with CDAD in a mean of 4.9 days, effective in 9 of 10 patients. However, stool *C. difficile *toxins persisted longer than diarrheal symptoms (mean, 8.1 days) for patients in the rifaximin group [28]. Similar results were observed in the group that received vancomycin 1 g/d for 10 days, with the exception of time to eradication of stool *C. difficile *toxin, which was significantly shorter for patients who received vancomycin (mean, 4.8 d; *P* < 0.005) [28]. The findings from this Italian study suggest the potential benefit of rifaximin for the treatment of CDAD, but the study is limited by small sample size and lack of posttreatment followup data. 

The current findings that hospitalized patients who received a 10-day course of rifaximin were asymptomatic during the long-term post treatment followup period of up to 261 days provide the first report that rifaximin may help prevent posttreatment CDAD recurrences when administered as first-line therapy. Two previous open-label studies that investigated the efficacy of rifaximin for prevention of CDAD recurrence focused on patients with recurrent CDAD who had received prior systemic antibiotic treatment [24, 25]. Rifaximin 1200 mg/d for ≥2 weeks followed by rifaximin 600 mg/d for 2 weeks resolved symptoms in a mean of 7.6 days and prevented recurrence for 1 month in 5 of 6 patients with recurrent CDAD [24]. In addition, 7 of 8 patients with recurrent CDAD who received rifaximin 400 to 800 mg/d for 2 weeks beginning immediately after successful treatment with vancomycin remained asymptomatic for 51 to 431 days after treatment [25]. 

The current study has several strengths relative to previously published investigations, including the lack of vancomycin or metronidazole treatment within 48 hours of rifaximin treatment, all but one of these patients had no history of CDAD, and the availability of long-duration post treatment followup data. However, several limitations warrant consideration, including the open-label design and small sample size. The small number of enrolled patients, which precluded randomization and comparison of rifaximin with standard therapies, is explained by difficulty recruiting patients who met the inclusion criteria. Study recruitment at the single hospital site depended on referrals from hospital physicians, many of whom treated diarrheal symptoms empirically before stool toxin test results were available, thus limiting patient eligibility. 

Despite these limitations, the current study provides supportive evidence that the nonabsorbed antibiotic rifaximin may be effective for the treatment of CDAD when administered as initial therapy in previously uninfected patients and may confer long-term protection against recurrence. Given that it has a favorable safety profile and a success rate comparable to published rates for vancomycin or metronidazole, rifaximin may offer a promising alternative to standard therapies for CDAD. An additional consideration for future studies would be related to cost of the therapy and the indirect cost savings of adherence to a well-tolerated therapy and prevention of recurrence. These encouraging results, along with the increasing incidence and severity of CDAD, suggest a need for further investigation. Randomized, controlled trials are warranted to further evaluate the efficacy of rifaximin relative to current standard therapies and to confirm the role of rifaximin in the treatment of CDAD.

## Figures and Tables

**Table 1:** Patient demographics and characteristics.

Patient	Age	Sex	Reason for hospitalization	Comorbidities	Previous CDAD
(1)	68	F	Deep vein thrombosis	Hypertension, CRI, nonsmall cell lung cancer, aortic valve repair, Hodgkin's lymphoma	No
(2)	62	F	Neutropenic fever and reinduction of chemotherapy	Acute myelogenous leukemia, hypertension, uterine cancer, history of VRE	Yes
(3)	68	F	Acute renal failure, volume depletion	Breast cancer, COPD, CHF, MGUS, Klebsiella pneumonia infection, anemia	No
(4)	41	M	CHF exacerbation (ejection fraction 15%)	Congenital cardiomyopathy; CRI	No
(5)	76	F	Pneumonia	hypertension, diabetes mellitus, ESRD	No
(6)	46	M	Diverticulitis requiring partial colectomy	Hypertension, umbilical hernia	No
(7)	39	M	Diarrhea, volume depletion	Right inguinal hernia	No
(8)	39	M	Failure to thrive	Extramedullary CML, graft-versus-host disease, pleural effusions, depression	No

CDAD: *Clostridium difficile*-associated diarrhea; CHF: chronic heart failure; CML: chronic myeloid leukemia; COPD: chronic obstructive pulmonary disease; CRI: chronic renal insufficiency; ESRD: end-stage renal disease; MGUS: monoclonal gammopathy of unknown significance; VRE: vancomycin-resistant *Enterococcus*.

**Table 2:** CDAD symptom improvement with rifaximin 1200 mg/day for 10 days.

Patient	Symptom resolution with rifaximin	TLUS, h*	Followup stool EIA^†^	Relapse at 2 weeks	Relapse (followup, d)
(1)	Yes	84	NA^‡^	No	No (261)
(2)	Yes^‡^	282	Positive	Yes	NA
(3)	Yes	128	Negative	No	No (194)
(4)	Yes	108	Negative	No	Yes (52)
(5)	Yes	187	Negative	No	No (162)
(6)	Yes	96	Positive	No	No (149)
(7)	Yes	192	Negative	No	No (160)
(8)	Yes	131	Negative	No	No (36)

CDAD: *Clostridium difficile*-associated diarrhea; EIA: enzyme immunoassay; NA: not applicable; TLUS: time to last unformed stool.

*From ingestion of first rifaximin tablet. ^†^Obtained on day 10 following initiation of rifaximin therapy. ^‡^Followup stool sample not provided. ^‡^Symptom resolution occurred 2 days after completing the 10-day course of therapy.
